# Supplementary material for: Vaccinated COVID-19 Index Cases Are Less Likely to Transmit SARS-CoV-2 to Their Household Contacts: A Cohort Study
Source: Vaccines (Basel). 2024 Feb 26;12(3):240. doi: 10.3390/vaccines12030240 (PMC10975059; doi:10.3390/vaccines12030240)
Supplement: Supplementary file 1 [file vaccines-12-00240-s001.zip › vaccines-2823257-supplementary.pdf]

Supplementary Materials:

Table S1. Household infection transmission effects of vaccination and previous SARS-CoV-2 infection his-tory in index cases and contacts

Variable	Infected contact n=87	Non-infected contact n=147	OR	95% CI	P value
**Index case vaccination y/o previous COVID-19 history**					
No	9	6	1.00	Reference	
Vaccination without previous COVID-19 history	52	84	0.41	0.14-1.23	0.111
previous COVID-19 history without vaccination	3	1	2.00	0.16-24.07	0.584
Vaccination and previous COVID-19 history	23	56	0.27	0.08-0.86	0.026
**Contact vaccination y/o previous COVID-19 history**					
No	3	5	1.00	Reference	
Vaccination without previous COVID-19 history	54	61	1.47	0.34-6.46	0.605
previous COVID-19 history without vaccination	2	6	0.56	0.06-4.76	0.591
Vaccination and previous COVID-19 history	28	75	0.66	0.13-2.78	0.534

CI: confidence interval; OR: odds ratio.

Supplementary Table S2. Multivariate logistic regression of factors associated with SARS-CoV-2 transmission to household contacts

Variable	aOR	95% CI	P value
**Age group (years)**			
0-17	1.00	Reference	
18-44	1.42	0.47-4.31	0.535
45-64	1.15	0.38-3.45	0.798
≥65	3.42	1.01-11.57	0.048
**Sex**			
Male	0.68	0.36-1.29	0.233
Female	1.00	Reference	
**Index case vaccination y/o previous COVID-19 history**			
No	1.00	Reference	
Vaccination without previous COVID-19 history	0.20	0.05-0.76	0.018
previous COVID-19 history without vaccination	1.00	0.07-13.92	0.995
Vaccination and previous COVID-19 history	0.23	0.06-0.87	0.030
**Contact vaccination y/o previous COVID-19 history**			
No	1.00	Reference	
Vaccination without previous COVID-19 history	1.01	0.16-6.09	0.989
previous COVID-19 history without vaccination	0.42	0.03-5.07	0.495
Vaccination and previous COVID-19 history	0.41	0.07-2.41	0.324
**Smoker**			
Yes	2.31	1.17-4.54	0.015
No	1.00	Reference	
**Cohabitation with partner**			
Yes	2.3	1.06-5.05	0.034
No	1.00	Reference	

aOR: adjusted odds ratio (according to the remaining variables in the table); CI: confidence interval.
